# Circulating Methylated *XAF1* DNA Indicates Poor Prognosis for Gastric Cancer

**DOI:** 10.1371/journal.pone.0067195

**Published:** 2013-06-27

**Authors:** Zhi-Qiang Ling, Ping Lv, Xiao-Xiao Lu, Jiang-Liu Yu, Jing Han, Li-Sha Ying, Xin Zhu, Wang-Yu Zhu, Xian-Hua Fang, Shi Wang, Yi-Chen Wu

**Affiliations:** 1 Zhejiang Cancer Research Institute, Zhejiang Province Cancer Hospital, Zhejiang Cancer Center, Hangzhou, Zhejiang, China; 2 Department of Pathology, Zhejiang Province Cancer Hospital, Zhejiang Cancer Center, Hangzhou, Zhejiang, China; 3 Department of Endoscopy, Zhejiang Province Cancer Hospital, Zhejiang Cancer Center, Hangzhou, Zhejiang, China; Vanderbilt University Medical Center, United States of America

## Abstract

**Background:**

Methylated DNA in fluids may be a suitable biomarker for cancer patients. *XAF1* has been shown to be frequently down-regulated in human gastric cancer (GC). Here, we investigated if *XAF1* methylation in GC could be a useful biomarker.

**Methods:**

Real-time RT-PCR was used to detect *XAF1* mRNA expression; immunohistochemistry and western blot were used to examine XAF1 protein expression in GC tissues (n = 202) and their corresponding para-cancerous histological normal tissues (PCHNTs). Real-time methylation specific-PCR was used to investigate *XAF1* promoter methylation in the same panel of GC tissues, their PCHNTs and sera.

**Results:**

We confirmed frequent *XAF1* down-regulation in both mRNA and protein levels in GC tissues as compared to normal controls and PCHNTs. *XAF1* hypermethylation was evidenced in 83.2% (168/202) of GC tissues and 27.2% (55/202) of PCHNTs, while no methylation was detected in the 88 normal controls. The methylation level in GC tissues was significantly higher than that in PCHNTs (*p<*0.05). The hypermethylation of *XAF1* significantly correlated with the down-regulation of *XAF1* in GC tissues in both mRNA and protein levels (*p*<0.001 each). Moreover, we detected high frequency of *XAF1* methylation (69.8%, 141 out of 202) in the sera DNAs from the same patients, while the sera DNAs from 88 non-tumor controls were negative for *XAF1* methylation. The *XAF1* methylation in both GC tissues and in the sera could be a good biomarker for diagnosis of GC (AUC = 0.85 for tissue and AUC = 0.91 for sera) and significantly correlated with poorer prognosis (*p*<0.001). In addition, after-surgery negative-to-positive transition of *XAF1* methylation in sera strongly associated with tumor recurrence.

**Conclusions:**

1) Dysfunction of *XAF1* is frequent and is regulated through *XAF1* promoter hypermethylation; 2) Detection of circulating methylated *XAF1* DNAs in the serum may be a useful biomarker in diagnosis, evaluating patient’s outcome (prognosis and recurrence) for GC patients.

## Introduction

Gastric cancer is one of the most common cancers in China, with a high incidence and mortality, approximately accounting for 10% of all malignancies [1]. Gastric tumorigenesis is a complicated, multiple-step process involving alterations of many genes [2]. Aberrant promoter methylation is one of the major mechanisms to silence some tumor suppressor genes and tumor related genes and plays very important roles in the pathogenesis and progression in human cancers [3], [4]–[6], including gastric cancer [7], [8]. Meanwhile, accumulating data strongly suggested that DNA methylation could be useful and powerful biomarker in cancer risk evaluation [5], [7], early diagnosis [7], predicting patients’ prognosis [7], [8], and evaluating the sensitivity to chemotherapeutic drugs [9]. Our recent study and other researches demonstrated that detecting circulating methylated DNA in blood is a potent and practical approach for cancer patients [7], [10], [11].

X-linked inhibitor of apoptosis (*XIAP*)-associated factor 1 (*XAF1*) is a novel negative regulator of *XIAP*, which reverses XIAP’s protection role on tumor cells [12]–[14]. The loss of *XAF1* expression will render tumor cells resistance to apoptosis and promote tumor cell survival [14]–[17]. Dysfunction of *XAF1* has been reported in several human cancers probably through promoter methylation [15]–[19], suggesting its importance in tumorigenesis. In human gastric cancer, *XAF1* has been reported to be frequently and significantly down-regulated and this down-regulation of *XAF1* probably through DNA hypermethylation of specific CpG sites [15], [16], [18]. However, no report is available about *XAF1* methylation in blood and its potentiality as a biomarker. In the present study, we examined *XAF1* promoter methylation in paired tissue and serum samples from a large panel of patients with gastric cancer, and evaluated circulating methylated *XAF1* as a potential biomarker for gastric cancer.

## Material and Methods

### Ethics Statement

De-identified human tissue samples and sera were obtained from Zhejiang Province Human Tissue Specimen Bank. The use of specimens was approved by the Institutional Review Board at Zhejiang Province Cancer Hospital. Written informed consent was obtained from each patient in accordance with the requirements of our institution’s board of ethics. 88 non-cancer volunteers provided written informed consent. Part of the specimens were from Zhejiang Province People’s Hospital and the First People’s Hospital of Chunan County. The Institutional Review Board on Medical Ethics of Zhejiang Province People’s Hospital and the First People’s Hospital of Chunan County approved the method of specimen collection including written informed consent from all patients, respectively.

### Tissue Specimen

Paired tumor and para-cancerous histological normal tissues (PCHNT) specimens were collected at the time of surgery from 202 patients with primary gastric adenocarcinoma at Zhejiang Province Cancer Hospital, Zhejiang Province People’s Hospital and the First People’s Hospital of Chunan County from January 2008 to December 2009. The PCHNT was assessed microscopically for the presence of normal cells and absence of dysplastic cells. None of these cases had undergone any medical treatment before surgery. Demographic, clinical and histopathological parameters of these cases were shown in Table 1. The growth pattern of tumor cells was determined according to Ming’s classification [20]. All recruited patients had been followed-up periodically until the due date. Antral mucosa biopsy specimens from 88 non-cancer volunteers by gastroscopy were randomly collected as controls within the same period, including 54 men and 34 women, with an average age of 52.9 years old. Among these volunteers, 48 patients were diagnosed with chronic non-atrophic gastritis. Meanwhile, paired serum samples were collected before surgery or endoscopy.

**Table 1:** Clinico-pathological correlations of XAF1 protein expression in gastric cancer tissues.

Clinicopathological parameters	Number of cases	XAF1 protein expression by IHC			?2 (*p*-values)
		High level	Low-moderate level	Absent	
Gender					
Male	120	23	52	45	1.276 (0.528)
Female	82	11	40	31	
Age at diagnosis					
<60	145	23	67	55	0.349 (0.840)
≥60	57	11	25	21	
Tumor location					
Cardia	63	11	26	26	0.712 (0.700)
Body/Antrum	139	23	66	50	
H. *pylori* infection					
Negative	102	25	47	30	10.923 (0.04)
Positive	100	9	45	46	
Tumor size					
<5cm	92	25	49	18	27.588 (*p*<0.0001)
≥5cm	110	9	43	58	
Growth pattern					
Expanding Type	82	12	39	31	0.520 (0.771)
Infiltration type	120	22	53	45	
Histological differentiation					
Well/moderate	121	29	54	38	12.285 (0.02)
Poor	81	5	38	38	
Lymphatic invasion					
Negative	140	34	76	30	54.507 (*p*<0.0001)
Positive	62	0	16	46	
Venous invasion					
Negative	148	30	73	45	13.293 (0.001)
Positive	54	4	19	31	
Invasive depth					
T1/T2	57	21	27	9	29.010 (*p*<0.0001)
T3/T4	145	13	65	67	
Lymph node metastasis					
N0	68	26	40	2	64.462 (*p*<0.0001)
N1–3	134	8	52	74	
Distant metastasis					
M0	184	33	92	59	31.575 (*p*<0.0001)
M1	18	1	0	17	
TNM stage					
Stage I/II	64	27	37	0	74.125 (*p*<0.0001)
Stage III/IV	138	7	55	76	

IHC: Immunohistochemistry; ISS: immunohistochemical staining score. High level: ISS 9∼12; Low/Mod level: ISS 1∼8; Absent: ISS 0.

### Analysis of Helicobacter Pylori (*H. pylori*) Infection

Biopsies were obtained from all patients who had endoscopic examination. *H*. *pylori* status was determined by rapid Urease test and Giemsa staining methods [21], [22]. It was considered as *H*. *pylori* infection when both tests were positive, and the samples with single positive were excluded for statistical analysis [22].

### Real-time RT-PCR

The mRNA expression of *XAF1* was analyzed by real-time RT-PCR [23]. Total RNAs were extracted using the Trizol (Gibco). A total of 3 µg total RNAs was subjected to reverse transcription using M-MLV reverse transcriptase (Promega). The glyceraldehyde phosphate dehydrogenase (*GAPDH*) was selected as the internal reference. The sequences of *XAF1* primers were as follows: (F) 5′-TGGGTGTAGGATTCTCCAGG-3′, (R) 5′- GGTTTGCCCAAG GACTACAA-3′. *GAPDH* primer sequences were as follows: (F) 5′-CATGA GAAGTATGACAACAGCCT-3′, (R) 5′-TAATTTTAGGTTAGAGGGTTATTGT- 3′. The 2^−ΔΔCt^ method was used to calculate relative changes in gene expression.

### Immunohistochemical Staining

The expression of XAF1 protein was determined by immunohistochemical analysis with XAF1 monoclonal antibody (Santa Cruz Biotechnology). Immunohistochemical staining for XAF1 was carried out using representative paraffin-embedded specimens from the 202 GC patients. After deparaffinization, antigen retrieval in 0.01 M citrate buffer, and inactivation of endogenous peroxidase activity in 3% H_2_O_2_/methanol, we incubated the slides with antibody for XAF1 at 4°C overnight, and immunohistochemical staining, following a standard avidinbiotin-peroxidase complex technique, was carried out using 3,3′-diaminobenzidine (DAB) as the chromogen. Nuclei were counterstained with hematoxylin. The immunohistochemical staining score (ISS) is determined by three independent pathologists combining staining frequency and intensity as follows [24], [25]: no staining is scored as 0, 1∼10% of cells stained scored as 1, 11∼50% as 2, 51∼80% as 3, and 81∼100% as 4. Staining intensity is rated on a scale of 0 to 3, with 0, negative; 1, weak; 2, moderate; and 3, strong. When there is multifocal immunoreactivity and there are significant differences in staining intensities between foci, the average of the least intense and most intense staining was recorded. The raw data were converted to the ISS by multiplying the frequency score and the staining intensity score. Theoretically, the ISS could range from 0 to 12. An ISS of 9∼12 was considered strong immunoreactivity, 5∼8 was considered moderate, 1∼4 was considered weak, and 0 was scored as negative. Sections in which the staining could not be well characterized were considered equivocal.

### Western Blot Analysis

Paired tumor and PCHNT specimens of 20 cases were randomly selected from 202 gastric cancer patients for western blot analysis. Total protein was extracted and then quantified using the Lowry method [26]. Western blot analysis was performed using anti-XAF1 monoclonal antibodies (Santa Cruz, CA) according to previous report [6]. β-tubulin was served as an internal control.

### DNA Extraction, Bisulfite Modification and Real-time Methylation Specific-PCR (MSP)

Serial 5-mm sections that contained carcinoma and non-neoplastic tissues were mounted on non-coated glass slides and dried at 37°C overnight. After deparaffinization and staining with hematoxylin and eosin (H&E), we collected 5,000 nuclei from 5 to 10 serial sections using a 27G needle. The collected nuclei were treated with 40 ml of 200 mg/ml proteinase K (Sigma-Aldrich Co., St. Louis, MO) at 42°C, for 72 hr. The paramagnetic bead technology (AxyPrep Mag Blood gDNA kit, Axygen Scientific, Inc., Union City, CA) was utilized to isolate genomic DNA from fresh blood according to kit’s protocol. The protocol consists of the following step: lysis, binding, washing, and elution. Contaminants are removed during the binding and washing steps. The quality of DNA was assessed by the A260/280 ratio at NanoDrop ND-1000 spectrophotometer (NanoDrop Technologies, Inc., Wilmington, DE), DNA integrity was checked by denaturing agarose gel electrophoresis.

DNAs were modified by sodium bisulfite using the EpiTect Bisulfite kit (Qiagen Inc.) following manufactory’s instructions. Modified DNAs were analyzed by real-time MSP on the ABI7500 PCR (ABI Co.) using the SYBR Premix Taq ExTaq Kit (TaKaRa Co. Ltd). *XAF1* methylation and unmethylation specific primers were designed as follows: *XAF1* (MF) 5′-TTTGTAAGAAACG AAATTTAATCGA-3′, (MR) 5′- CCTACCCTTAAAACCCACGAT-3′; *XAF1*(UF) 5′-TTTGTAAGAAATGAAATTTAATTGA-3′, (UR) 5′-CTCCTACCCTTAAAACC CACAAT-3′ [23]. Human genomic DNA (NEB) treated by SssI methyltransferase in vitro was used as a positive control. A peripheral blood DNA from a healthy subject was used as a negative control. The percentage of methylated DNAs in the samples were calculated according to the references as previous described [27], [28]. Methylated DNAs index was scored according to the percentage of DNA methylation; 0, <20%; 1, 20%−40%; 2, 40%−60%; 3, 60%−80%; and 4, >80%. The index score of 0 is considered as DNA unmethylation and scores 1–4 were considered hypermethylated, respectively [7], [27]. The 20% cut off threshold for DNA hypermethylation was based on control normal samples and internal quality controls obtained in the real-time MSP analysis.

### Cell Culture and Drugs Treatment

Human gastric cancer cell lines (AGS and KATO-III) were cultured in RPMI1640 medium supplemented with 10% fetal bovine serum, 100 U/mL penicillin, and 100 U/mL streptomycin at 37°C and 5% CO_2,_ respectively. Cultured cells were treated with 5-aza-2′-deoxycytidine (5-Aza-CdR) (Sigma-Aldrich) at a final concentration of 1.0 µmol/L. Trichostatin A (TSA) (Sigma-Aldrich) at a final concentration of 20 ng/ml was administrated following 5-Aza treatment or alone for 24 h. Cells were collected and subjected to DNA, RNA and protein purification and subsequent analyses.

### Statistical Analysis

SPSS 17.0 statistical software was adopted for data analysis. Counting data comparisons between groups were subjected to the *χ* ^2^ test and Fisher’s exact test. Survival analysis was computered by means of the Kaplan-Meier method and significant levels were assessed by means of the log-rank test. A univariate analysis with the Cox regression model was used to determine identified prognostic factors, and multivariate analysis with the Cox regression model was used to explore combined effects. For all statistical analyses, *p* values <0.05 were considered to be statistical significance.

## Results

### Down-regulation of *XAF1* in Primary Gastric Tumors

**Figure 1:**
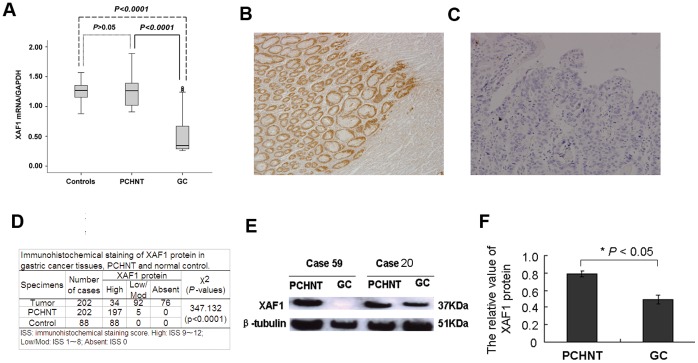
*XAF1* gene expression was down-regulated in gastric cancer (GC) tissues. **A**, *XAF1* mRNA expression level in GC tissues, PCHNTs (para-cancerous histological normal tissue) and non-cancer controls were determined by real-time RT-PCR and was normalized to *GAPDH*. **B**–**F**, XAF1 protein expression was down-regulated in gastric cancer tissues. **B**, a representative positive, high expression of XAF1 protein in a PCHNT tissues; **C**, Absent of XAF1 expression in a poorly differentiated GC; original magnification ×200. **D**, a summary of XAF1 immunohistochemical staining results in 202 gastric cancers. **E**, Representative western blot analysis of XAF1 expression in gastric cancer and corresponding PCHNT specimens with different *XAF1* methylation levels. PCHNT tissues in Case 59 (XAF1 methylation score: 0); GC tissues in Case 59 (methylated *XAF1* score: 4); PCHNT tissues in Case 20 (methylated score: 0); GC tissues in Case 20 (methylated *XAF1* score: 1). Beta-tubulin was used as internal control. **F**, Summary of the western blotting results from 20 GCs and corresponding PCHNTs presented as relative bands density normalized to the beta-tubulin of the same samples.

To investigate *XAF1* gene expression profile, we examined mRNA expression of *XAF1* in 88 non-cancer volunteers, and 202 primary gastric cancer tissues and their corresponding PCHNTs. As shown in Figure 1A, the *XAF1* expression was significantly reduced in gastric cancer samples as compared with that in normal controls and PCHNTs (*p<*0.001). However, there was no significant difference in *XAF1* expression in PCHNTs as compared to non-cancer controls.

Furthermore, *XAF1* expression was significantly lower in advanced tumors (stage III/IV) than that in early stage tumors (stage I/II) (*p<*0.0001, Figure S1A), and was significantly lower in poorly differentiated tumors than that in well/moderately differentiated tumors (*p<*0.0001, Figure S1B).

To check XAF1 protein level in gastric cancer tissues, we performed immunohistochemical analysis in the 202 gastric cancer tissues and their corresponding PCHNTs. Nuclear XAF1 expression was consistently present in the normal gastric epithelia showing high immunoreactive scores. The XAF1 protein expression was detected in high level in 97.5% (197/202) of PCHNTs (Figure 1B). However, high expression of XAF1 protein was only detected in 16.8% (34/202) of gastric cancer tissues; majority of gastric cancer tissues were absent or at a low level of XAF1 protein (Figure 1C). The immunohistochemical findings are summarized in Table 1. The immunohistochemical staining score in gastric cancer tissues was significantly lower than that in PCHNTs or in normal controls (*p<*0.0001, Figure 1D). The loss of XAF1 protein significantly correlated with *H. pylori* infection, tumor size, histological differentiation, lymphatic invasion, venous invasion, invasive depth, lymph node metastasis, distant metastasis and clinical stage (Table 1) (all *p<*0.05).

The immunohistochemical results of 20 gastric cancer tissues were further confirmed by means of western blot analysis. The representative western blotting results in two cases were shown in Figure 1E. The relative quantity of XAF1 protein expression was normalized to the β-tubulin of the same samples. The average XAF1 protein level in 20 gastric cancer tissues was significantly lower than that in PCHNTs (*p*<0.05) (Figure 1F).

### Promoter Hypermethylation of *XAF1* in Primary Gastric Tumors

**Figure 2:**
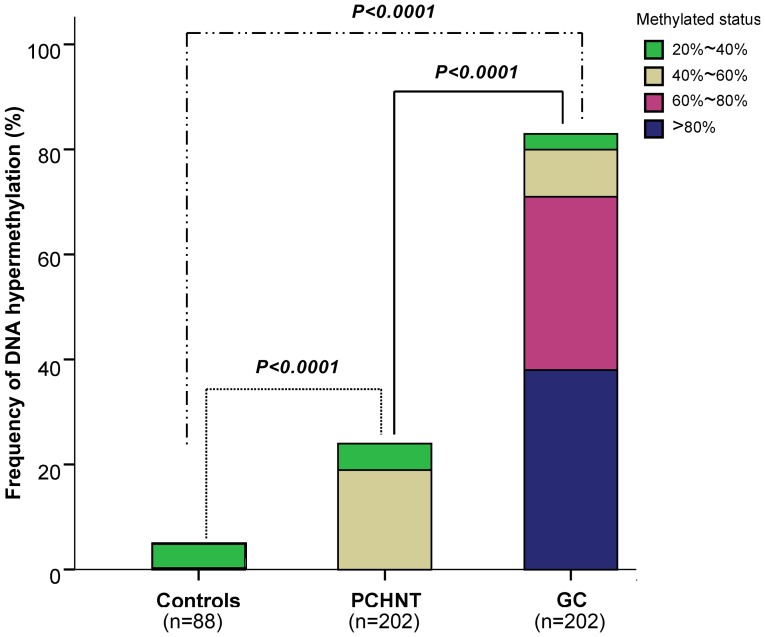
Summary of *XAF1* methylation in 202 gastric cancers tissues, 202 para-cancerous histological normal tissues (PCHNTs) from the same patients and 88 non-cancer volunteers. Data shows the frequency of *XAF1* hypermethylation (DNA methylation level ≥20%) in each group.

To investigate the molecular mechanisms for the *XAF1* silence in gastric cancers, we applied a real-time MSP technology to study the DNA methylation status of *XAF1* promoter. The frequency of *XAF1* hypermethylation in gastric cancer tissues, corresponding PCHNTs and non-cancer controls were 83.2% (168/202), 24.8% (50/202) and 5.7% (5/88), respectively. The hypermethylation frequency of *XAF1* in cancers is significantly higher than that in PCHNTs and non-cancer controls (all *p*<0.0001, Figure 2).

Of note, the methylated status of *XAF1* significantly correlated with some clinico-pathological parameters in gastric cancer, such as lymph node metastasis, T-stage, *H*. *pylori* infection, etc (all *p<*0.05, Table 2). No significant correlation between the hypermethylation of *XAF1* and gender, age at diagnosis, tumor site and distant metastasis was evidenced (all *p*>0.05) (Table 2).

**Table 2:** Clinico-pathological correlations of *XAF1* promotor hypermethylation in gastric cancer tissues and in sera.

Clinicopathological parameters	Number of cases	*XAF1* in tissues		?2 (*p*-values)	*XAF1* in serum		?2 test (*p*-values)
		M	U		M	U	
Cases							
Tumor	202	168	34	207.041 (*P<*0.0001)	141	61	283.742 (*P<*0.0001)
PCHNT	202	50	152				
Normal control	88	5	83		0	88	
Gender							
Male	120	97	23	1.151 (0.34)	83	37	0.057 (0.877)
Female	82	71	11		58	24	
Age at diagnosis							
<60	145	122	23	0.345 (0.539)	105	40	1.663 (0.234)
≥60	57	46	11		36	21	
Tumor site							
Cardia	63	52	11	0.026 (0.842)	43	20	0.104 (0.744)
Body/Antrum	139	116	23		98	41	
H. pylori							
Negative	102	77	25	8.677 (0.004)	62	40	7.949 (0.006)
Positive	100	91	9		79	21	
Tumor size							
<5cm	92	67	25	12.909 (0.001)	42	50	46.744 (*P<*0.0001)
≥5cm	110	101	9		99	11	
Growth manner							
Expanding Type	82	70	12	0.476 (0.568)	60	22	0.743 (0.437)
Infiltration type	120	98	22		81	39	
Histological differentiation							
High/Medium	121	92	29	10.975 (0.001)	71	50	17.715 (*P<*0.0001)
Low	81	76	5		70	11	
Lymphatic invasion							
Negative	140	106	34	18.104 (*P<*0.0001)	80	60	34.678 (*P<*0.0001)
Positive	62	62	0		61	1	
Venous invasion							
Negative	148	118	30	4.676 (0.034)	93	55	12.738 (*P<*0.0001)
Positive	54	50	4		48	6	
Invasive depth							
T1/T2	57	36	21	22.714 (*P<*0.0001)	16	41	65.606 (*P<*0.0001)
T3/T4	145	132	13		125	20	
Lymph node metastasis							
N0	68	42	26	33.546 (*P<*0.0001)	17	51	97.612 (*P<*0.0001)
N1–3	134	126	8		124	10	
Distant metastasis							
M0	184	151	33	1.795 (0.319)	151	33	1.795 (0.319)
M1	18	17	1		17	1	
TNM stage							
Stage I/II	64	37	27	43.025 (*P<*0.0001)	11	53	123.032 (*P<*0.0001)
Stage III/IV	138	131	7		130	8	

M: methylation; U: Unmethylation.

### *XAF1* Promoter Hypermethylation is Associated with its Transcriptional Silencing in Gastric Cancer Cells

**Figure 3:**
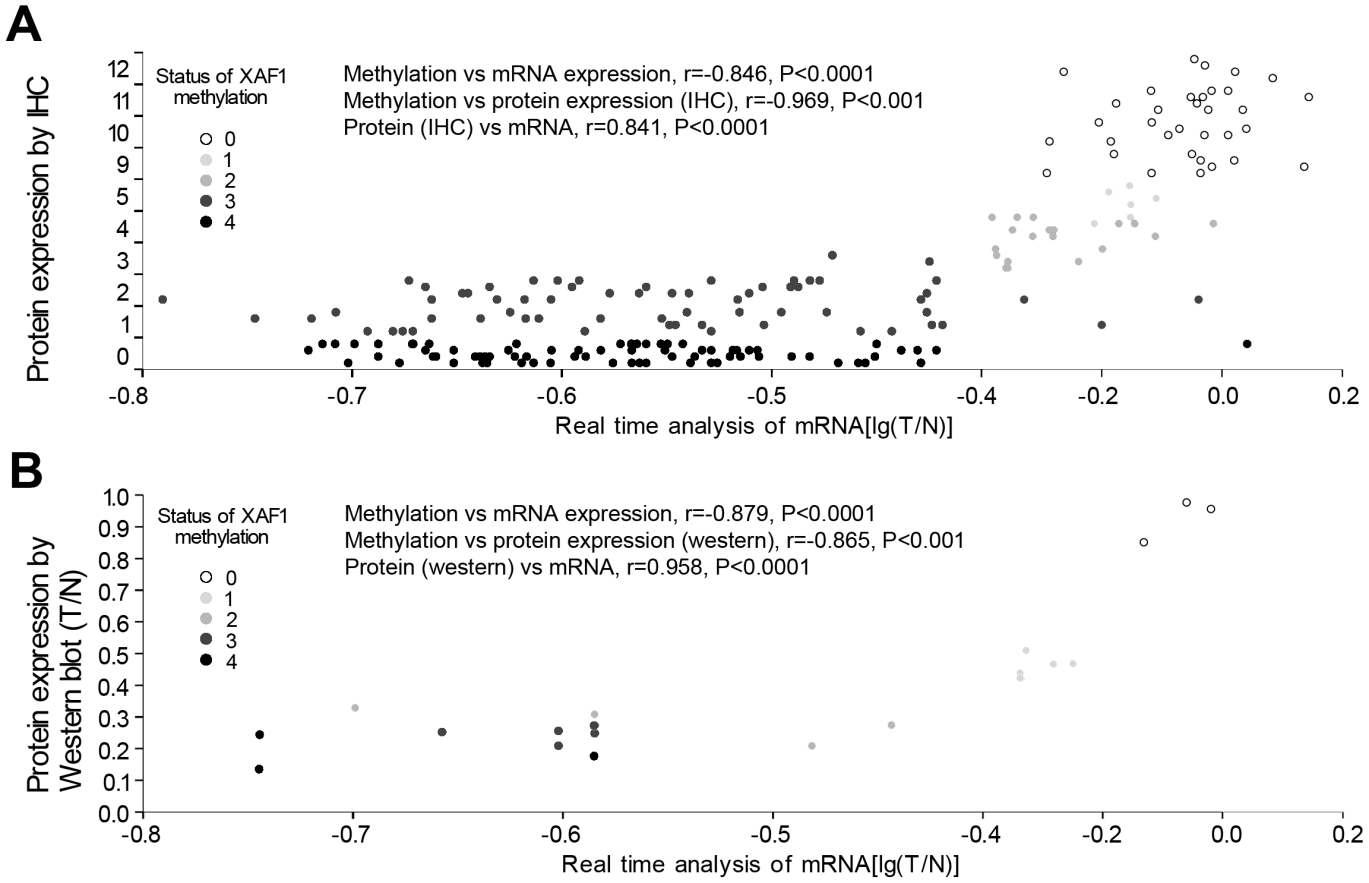
Correlation of *XAF1* methylation with *XAF1* expression. **A**, Correlation of *XAF1* methylation with *XAF1* mRNA level determined by RT-PCR analysis and XAF1 protein expression determined by immunohistochemical analysis in 202 gastric cancer tissues. **B**, Correlation of *XAF1* methylation with *XAF1* mRNA level determined by RT-PCR analysis and XAF1 protein expression determined by western blotting analysis in 20 frozen gastric cancer tissues. In both **A** and **B**, *XAF1* methylation scores inversely correlated with *XAF1* gene expression in both mRNA and protein levels.

To examine the relationships between *XAF1* methylation and *XAF1* expression, we compared the *XAF1* methylation level with *XAF1* mRNA level and protein levels determined either by immunohistochemical analysis (Figure 3A) or western blotting (Figure 3B) by the Spearman correlation analysis. As shown in Figure-3A, XAF1 protein expression (determined by immunohistochemical analysis) in 202 GC tissues was closely correlated with *XAF1* mRNA level [lg(T/N)] (determined by RT-PCR) (*γ* = 0.841, *p<*0.0001). More important, *XAF1* methylation level was significantly associated with *XAF1* mRNA level (*γ* = - 0.846, *p<*0.0001) and XAF1 protein level (*γ* = −0.969, *p<*0.0001). Similar results were obtained when analyzing the correlation of *XAF1* promoter methylation and the XAF1 protein expression determined by western blot analysis (Figure 3B). These results strongly indicted that *XAF1* expression is regulated by *XAF1* promoter methylation.

### 5-Aza-CdR Administration Restores the Expression of *XAF1*

**Figure 4:**
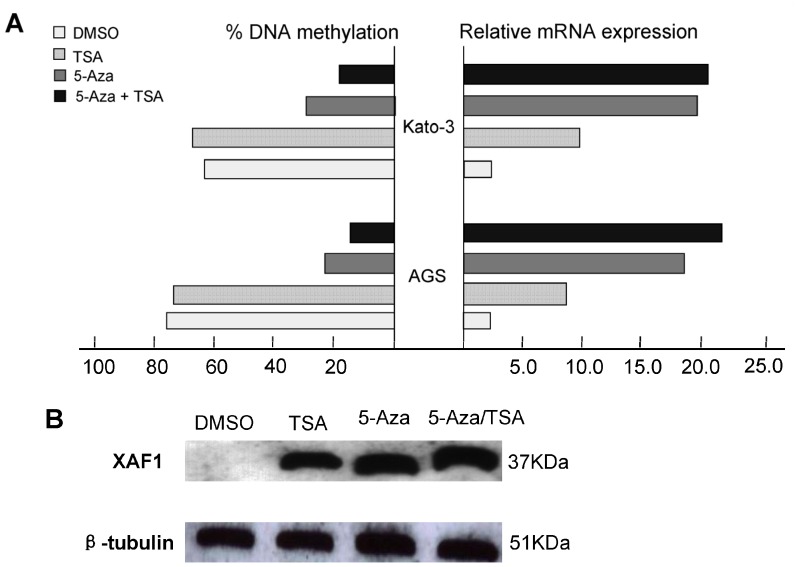
A: Transcriptional level of *XAF1* gene in gastric cancer cell lines following the 5-Aza-CdR or/and TSA treatments. Two gastric cancer cell lines (AGS and KATO-III) were treated with 1.0 µmol/L 5-Aza-CdR for 72 hours and/or 100 nM TSA for 24 hours. The methylation levels were determined by real-time MSP. We performed real-time RT-PCR analysis in triplicate for each cDNA sample and used median values in three experiments. The relative *XAF1* mRNA expression was normolized to the *GAPDH* of the same samples using the formula 2^−ΔΔCT^. The results were multiplied by 100 for a better visualization. The percentage of *XAF1* DNA methylation is shown on the left side; whereas the relative mRNA expression of *XAF1* is shown on the right side. **B**: Expression of XAF1 at the protein level following 5-Aza-CdR and TSA treatments. Western blot analysis of AGS cells following treatment with DMSO (control), 5-Aza-CdR, or 5-Aza-CdR/TSA for 72 hours demonstrate up-regulation of the XAF1 proteins in treated cells as compared to control (DMSO). The beta-tubulin is shown as a loading control.

To further confirm the epigenetic regulation of *XAF1* expression, AGS and KATO-III cells were treated with 5-Aza-CdR and/or TSA. *XAF1* mRNA expression was reactivated in both gastric cancer cell lines, accompanied by demethylation of *XAF1* promoter (Figure 4), indicating that *XAF1* is transcriptionally silenced in these cells by DNA hypermethylation. Interestingly, TSA treatment alone was effective in restoring *XAF1* expression in AGS and KATO-III without significant change of *XAF1* methylation level, suggesting that histone modifications may also be involved in regulating *XAF1* expression. However, administration of TSA following 5-Aza-CdR had an additive effect in restoring gene expression with a further decrease in the methylation level of *XAF1*. These results are in agreement with recent studies that suggested that TSA can have a demethylation effect in a gene-specific manner [5]. The western blot analysis using AGS cells, as a model, confirmed the up-regulation of XAF1 proteins following the 5-Aza-CdR and 5-Aza-CdR/TSA treatments (Figure 4).

### Detection of Circulating *XAF1* Methylation

**Figure 5:**
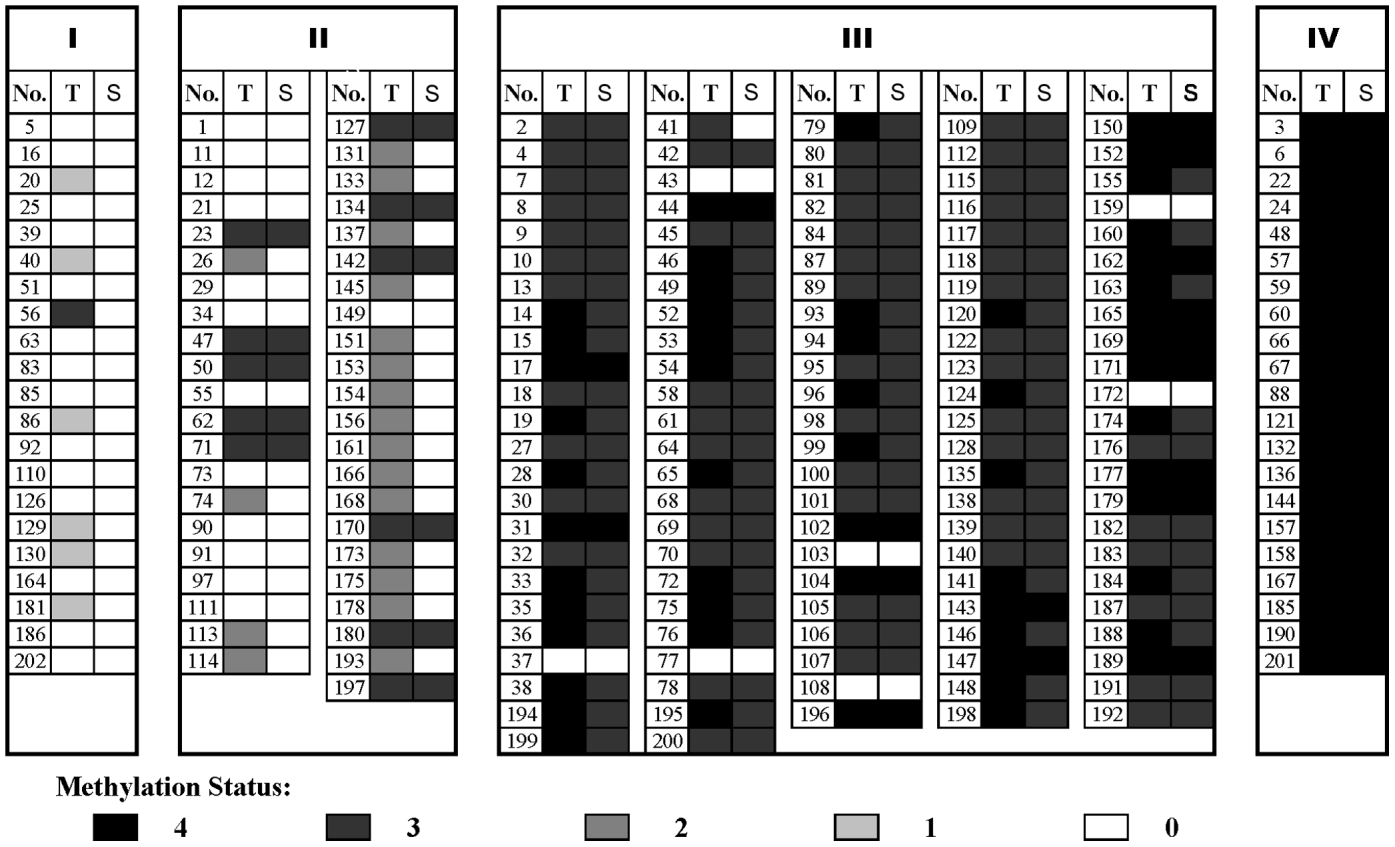
Compasion of *XAF1* methylation between in GC tissues and in corresponding peripheral blood (serum), T: tumor tissues; S: peripheral blood (serum).

We detected high frequency of *XAF1* methylation in primary gastric cancer (83.2%), suggesting that it may be a good biomarker if *XAF1* methylation could be detectable in serum. Therefore, we examined *XAF1* methylation in the matched serum DNAs from the 202 GC patients. *XAF1* methylation was detected in 141 (69.8%) serum DNAs from the 202 gastric cancer patients (Figure 5). In contrast, *XAF1* methylation was not detected in serum DNAs from the 88 non-cancer controls.

Next, we confirmed the consistency in *XAF1* methylation between tumor tissues and corresponding serum. In 168 cases that showed *XAF1* methylation in tumor tissues, 141 displayed *XAF1* methylation in their paired serum, giving a consistency of 83.9% between them. And in the 34 gastric cancer patients without *XAF1* methylation in their gastric cancer tissues, no methylation was found in all the serum DNAs (Figure S2).

### *XAF1* Methylation as a Biomarker in the Diagnosis of Gastric Cancer

**Figure 6:**
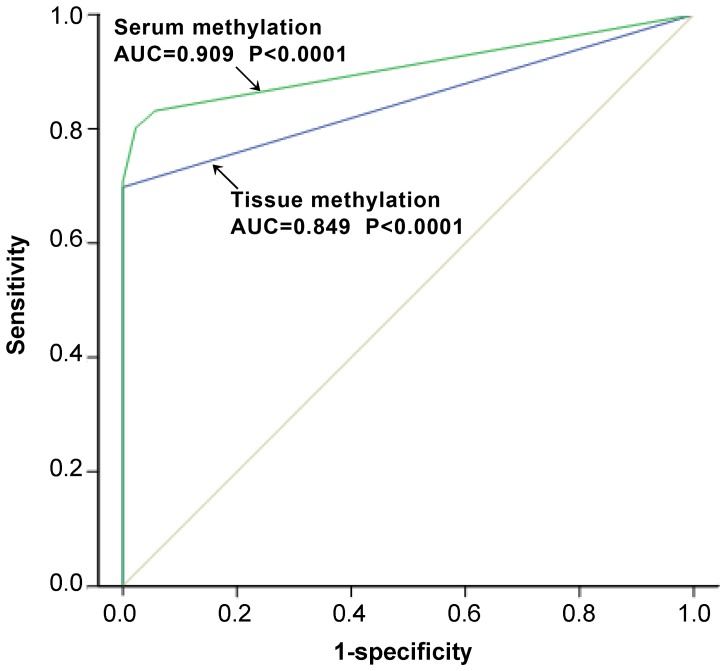
*XAF1* methylation as a biomarker for diagnosis of gastric cancer. Receiver operating characteristic (ROC) curve analysis was used to evaluate the possibility of *XAF1* methylation in gastric cancer tissues or in serum as a biomarker to diagnose gastric cancer. For *XAF1* methylation in gastric cancer tissues, an area under the ROC curve (AUC) is 0.849 (95% confidence interval, 0.806–0.892; *P<*0.0001). For *XAF1* methylation in serum, the AUC is 0.909 (95% confidence interval, 0.875–0.942; *P<*0.0001).

To evaluate the value of *XAF1* methylation as a biomarker in diagnosing GC, we plotted receiver operating characteristic (ROC) curves and calculated area under the curve (AUC) values using DNA methylation data from both GC tissues and sera. Both the ROC analyses of *XAF1* methylation in tissues and sera revealed significant discriminative capacity (Figure 6); the AUC value of tissues *XAF1* methylation was 0.849 (95% confidence interval, 0.806–0.892; *p*<0.0001), the AUC value of serum *AXF1* methylation was 0.909 (95% confidence interval, 0.875–0.942; *p*<0.0001).

In addition, like *XAF1* methylation status in gastric cancer tissues and the sera significantly correlated with lymph node metastasis, T-stage, clinical stage, and other clinico-pathological parameters (all *p<*0.05, Table 2).

### *XAF1* Methylation Correlated with Prognosis of Gastric Cancer Patients

**Figure 7:**
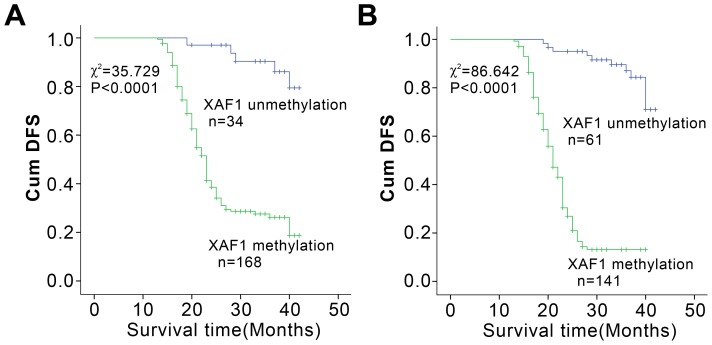
*XAF1* methylation in gastric cancer tissues and sera correlated with patients’ prognosis. Cumulative disease-free survival (Cum DFS) curves are plotted agains*t XAF1* DNA methylation level in gastric cancer tissues (A) and in the sera (B). In both A and B, Kaplan-Meier analysis were used and *P<*0.0001, respectively.

The significant correlation of *XAF1* methylation with many clinico-pathological parameters (Table 2) suggested that it may associate with the prognosis of gastric cancer patients. Until the due date of Follow-up, 113 of 168 patients with *XAF1* hypermethylation in gastric cancer tissues went rapid disease progression or died. The Median disease free survival (DFS) was only 23.4 months. In contrast, in the 34 patients without *XAF1* hypermethylation in their gastric cancer tissues, only 5 patients were deteriorating, and the Median DFS was 39.6 months. Kaplan-Meier analysis demonstrated that patients with *XAF1* hypermethylation in their gastric cancer tissues exhibited an obvious worse survival than that without *XAF1* hypermethylation (*p<*0.0001) (Figure 7A). Similarly, Kaplan-Meier analysis proved that patients with positive serum *XAF1* methylation had significantly lower DFS than that in the patients without serum *XAF1* methylation (*p*<0.0001) (Figure 7B), indicating that *XAF1* promoter methylation in serum was an unfavorable predictor for the gastric cancer patients.

Cox regression analysis revealed that *XAF1* methylation in sera is an independent factor on patients’ survival: patients with *XAF1* methylation had worse prognosis (*p<*0.0001; Hazard ratio, 5.710; 95% CI, 3.474∼9.383). In addition, TNM stages and age at diagnosis could be considered as the influencing factor of prognosis in gastric cancer, only when the effect of *XAF1* methylation was eliminated. Moreover, circulating methylated *XAF1* in blood had a greater impact on the prognosis than that in TNM stages (Table S1).

### Positive Transition of Circulating *XAF1* Methylation Predicts Tumor Recurrence and Poor Prognosis

Among the 202 patients who were evaluated for preoperative circulating *XAF1* methylation, 72 patients received follow-up examinations of circulating *XAF1* methylation 2∼5 times at intervals of 1∼6 months after surgery. Among 12 recurrent patients, 10 patients displayed negative-to-positive transition in *XAF1* methylation status in their sera, the other two showed always positive for *XAF1* methylation, suggesting that monitoring of *XAF1* methylation in serum may be a good marker for predicting tumor recurrence (Table 3).

**Table 3:** Circulating *XAF1* methylation change and patients outcomes.

The outcome of patients	Circulating *XAF1* methylation, after surgery			Total
	Negative always	N to P	Positive always	
DFS	39	0		39
Recurrence		10	2	12
Death			21	21
Total	39	10	23	72

DFS: disease-free survival.

N to P: Circulating *XAF1* methylation changed from negative to positive in the follow-up after surgery therapy.

## Discussion

*XAF1* (*XIAP*-associated factor 1) is a novel *XIAP* binding protein which could disturb the combining of *XIAP* with caspases, abolishes its protection on tumor cells and results in tumor cell apoptosis [12]–[14]. The anti-apoptosis function of *XIAP* is determined by the ratio of expression levels of *XIAP* against *XAF1* [15]. Reduced expression or silence of *XAF1* is a frequent event in human tumors [16]–[19]. In the present study, we first confirmed that *XAF1* gene expression was significantly down-regulated in both mRNA level and protein level in a large panel of primary gastric cancer tissues, and the down-regulation of *XAF1* expression was significantly associated with tumor stages, metastasis and so on, implicating loss of *XAF1* function in tumor progression. This is consistent with other research reports [17]–[19]. To explore the molecular mechanisms responsible for the *XAF1* silence, we examined *XAF1* promoter methylation in a large panel gastric cancer tissues (*n* = 202) and normal controls (*n* = 88) using a real-time MSP technology. We detected a high frequency (83.2%) of DNA hypermethylation of *XAF1* in gastric cancer tissues. The DNA hypermethylation of *XAF1* significant correlated with the down-regulation of *XAF1* in both mRNA and protein levels in gastric cancer tissues (Figure 3). In addition, 5-Aza-CdR treatment significantly restored *XAF1* expression in *XAF1* silenced gastric cancer cell lines (Figure 4). These data strongly suggest that frequent down-regulation of *XAF1* in gastric cancer cells is regulated by its promoter hypermethylation. Interestingly, treatment of gastric cancer cells with TSA, a histone deacetylase inhibitor also restored *XAF1* expression, alone or combined with 5-Aza-CdR treatment, indicating that histone modification may also be involved in *XAF1* regulation.

DNA methylation may be used as a potential tumor biomarker in various human cancers [3]–[11], [23]. The presence of cell-free DNA circulating in peripheral blood has been described in patients with malignant processes, and active release of tumor DNA into the circulation has been reported [29]–[31]. Numerous studies have demonstrated tumor-specific alterations in circulating DNAs recovered from plasma or serum of patients with various malignancies that match genetic changes present in primary tumors, a finding that has potential for molecular diagnosis and prognosis [29]–[32]. The nucleic acid markers described in circulating DNAs include oncogene mutations, microsatellite alterations, gene rearrangements, and epigenetic alterations, such as aberrant promoter hypermethylation [7], [8], [30]–[34]. On the basis of these observations, especially we detected a high frequency of *XAF1* DNA methylation in gastric cancer tissues (83.2%), we decided to explore the possibility to use *XAF1* promoter methylation in the serum as a biomarker in gastric cancer patients. First we demonstrated a great consistency in detecting *XAF1* methylation between sera and primary gastric cancer tissues. Next we analyzed the possibility of using circulating methylated *XAF1* as a diagnostic marker using ROC analysis. The AUC (area under curve) of 0.909 demonstrated a high diagnostic value of detecting *XAF1* methylation in serum samples. Therefore, our data suggest that detection of *XAF1* methylation in circulating DNA could be used as a non-invasive biomarker for diagnosis of gastric cancer. In addition, our results also showed that positive *XAF1* methylation in serum was an independent prognostic factor; predicting poor prognosis. More interestingly, transition from negative to positive of circulating *XAF1* methylation after surgery was significantly associated with tumor recurrence. These data indicate that detection of *XAF1* methylation in circulating serum DNA also can be a tumor biomarker for predicting the gastric cancer patients’ prognosis and for monitoring the tumor recurrence after surgery treatment. Because the number of the patients underwent this follow-up serum examination was small, further research in a large sample size of patients’ number is needed to confirm this interesting finding and to optimize the strategy and protocol for this purpose. In any event, comparing with the traditional ways such as gastroscopy examination for gastric cancer patients, the most remarkable characteristic of circulating methylated *XAF1* detection is efficient, rapid, low cost, non-invasive, not destroying environment, high diagnostic conformable rate and so on, and as such demonstrate important application prospects in the future.

### Conclusions

Dysfunction of *XAF1* is frequent and is regulated through *XAF1* promoter hypermethylation in gastric cancer. Circulating methylated *XAF1* DNA was associated with tumor burden and malignant progression, which may be a valuable biomarker for diagnosis of gastric cancer, predicting patients’ prognosis and monitoring tumor recurrence after surgery treatment.

## Supporting Information

Figure S1 jpg. **A**, *XAF1* gene expression level was lower in advanced gastric cancer (Stage III/IV) than that in early gastric cancer (Stage I/II). **B**, *XAF1* gene expression level was lower in poorly differentiated gastric cancer than that in well/moderately (well/Mod) differentiated gastric cancer, *p<*0.05.
(TIF)

Figure S2 jpg. Correlation analysis of *XAF1* hypermethylation results between GC tissues and paired peripheral blood (sera).
(TIF)

Table S1 xls. Multivariate survival analysis of clinico-pathological data of 202 gastric carcinoma cases. Cox analysis showed that that *XAF1* methylation in sera is an independent factor on patients’ survival (*p<*0.0001; Hazard ratio, 5.710; 95% CI, 3.474∼9.383). In addition, TNM stages and age at diagnosis could be considered as influencing factor of prognosis for patients with GC.
(DOC)
