# Supplementary figures and images for: Circulating Methylated XAF1 DNA Indicates Poor Prognosis for Gastric Cancer
Source: PLoS One. 2013 Jun 27;8(6):e67195. doi: 10.1371/journal.pone.0067195 (PMC3695092; doi:10.1371/journal.pone.0067195)

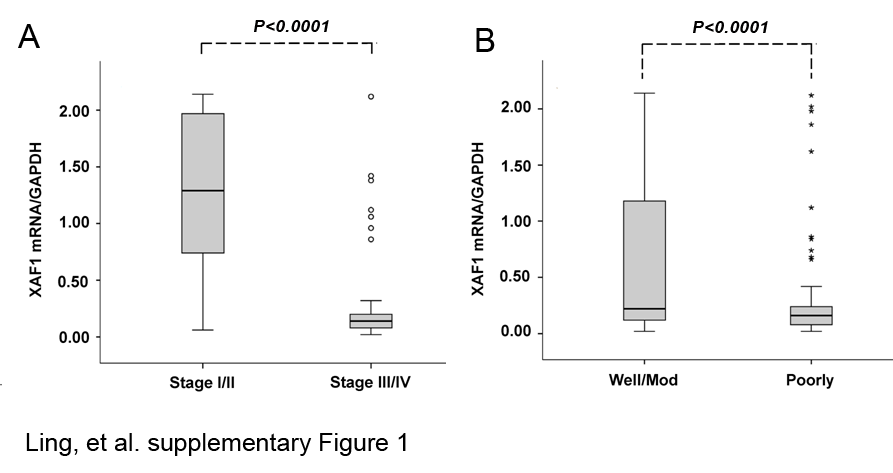

Supplement: Figure S1 — jpg. A, XAF1 gene expression level was lower in advanced gastric cancer (Stage III/IV) than that in early gastric cancer (Stage I/II). B, XAF1 gene expression level was lower in poorly differentiated gastric cancer than that in well/moderately (well/Mod) differentiated gastric cancer, p<0.05. (TIF) [file pone.0067195.s001.tif]
