# Supplementary material for: Circulating Methylated XAF1 DNA Indicates Poor Prognosis for Gastric Cancer
Source: PLoS One. 2013 Jun 27;8(6):e67195. doi: 10.1371/journal.pone.0067195 (PMC3695092; doi:10.1371/journal.pone.0067195)
Supplement: Table S1 — xls. Multivariate survival analysis of clinico-pathological data of 202 gastric carcinoma cases. Cox analysis showed that that XAF1 methylation in sera is an independent factor on patients’ survival (p<0.0001; Hazard ratio, 5.710; 95% CI, 3.474∼9.383). In addition, TNM stages and age at diagnosis could be considered as influencing factor of prognosis for patients with GC. (DOC) [file pone.0067195.s003.doc]

| Table S1：Multivariate survival analysis of clinicopathologic data of 202 gastric carcinoma cases | | | |
| --- | --- | --- | --- |
| Variables | Hazard ratio | 95% confidence interval | P-value |
| Gender (Male/Female) | 1.081 | 0.790-1.481 | 0.625 |
| Age at diagnosis (<60/≥60) | 0.644 | 0.454-0.913 | 0.014 |
| Tumor location (Cardia/Body,Antrum) | 0.963 | 0.665-1.395 | 0.842 |
| H. *pylori* infection (+/-) | 0.798 | 0.585-1.089 | 0.154 |
| Tumor size (<5cm/≥5cm) | 1.286 | 0.896-1.846 | 0.173 |
| Growth pattern (Expanding/Infiltration） | 1.025 | 0.738-1.423 | 0.883 |
| Histological differentiation （Well, Moderate/Poor） | 1.031 | 0.736-1.443 | 0.860 |
| Lymphatic invasion (+/-) | 0.703 | 0.465-1.063 | 0.095 |
| Venous invasion (+/-) | 1.239 | 0.816-1.879 | 0.314 |
| Invasive depth （T1,T2/T3,T4) | 0.902 | 0.579-1.405 | 0.649 |
| Lymph node metastasis (+/-) | 1.223 | 0.603-2.480 | 0.577 |
| Distant metastasis (+/-) | 1.958 | 0.929-4.129 | 0.078 |
| TNM stage (I,II/III,IV) | 0.013 | 0.005-0.037 | p<0.0001 |
| XAF1 in tumor tissues （M/U) | 1.331 | 0.789-2.246 | 0.284 |
| XAF1 in serums （M/U) | 5.710 | 3.474-9.383 | p<0.0001 |
